# Estimates of Prevalence of Pulmonary Hypertension according to Different International Definitions

**DOI:** 10.1155/2021/1385322

**Published:** 2021-11-28

**Authors:** Richa Tyagi, Surya Kant, Akshyaya Pradhan, Anupam Wakhlu, Darshan Kumar Bajaj, Jyoti Bajpai

**Affiliations:** ^1^Department of Respiratory Medicine, King George's Medical University, Lucknow, UP, India; ^2^Department of Cardiology, King George's Medical University, Lucknow, UP, India; ^3^Department of Rheumatology, King George's Medical University, Lucknow, UP, India

## Abstract

**Background:**

Pulmonary hypertension is a dreaded disease associated with considerable morbidity and mortality. The pulmonary hypertension developing due to chronic respiratory disease is a unique subset with symptoms often getting masqueraded by the underlying respiratory condition. The importance of early detection of this complication has been realized worldwide, and recently, the definition of pulmonary hypertension was revised to set the cutoff of mean pulmonary artery pressure (mPAP) at 20 mmHg instead of 25 mmHg at rest. In our study, we have tried to estimate the difference this new definition brings to the prevalence of pulmonary hypertension among interstitial lung disease patients at our centre.

**Methods:**

This was a cross-sectional study in which all the patients of ILDs (*n* = 239) attending the outdoor and indoor Department of Respiratory Medicine, King George's Medical University, India, for the duration of one year were subjected to transthoracic echocardiography along with measurement of serum pro-B-type natriuretic peptide (BNP) and troponin T values. The data were analyzed using the different definitions, and the prevalence was compared.

**Result:**

Incidence of pulmonary hypertension among ILD patients at mPAP cutoff ≥ 25 was 28.9%, while that at value ≥20 mmHg, incidence of pulmonary hypertension increased to 46.0%. An increment of 15–20% in incidence of pulmonary hypertension was observed among different types of ILD when cutoff of mPAP was changed.

**Conclusion:**

The new definition helps in a significant increase in the detection of pulmonary hypertension, which certainly helps in earlier detection and better management of patients.

## 1. Introduction

Pulmonary hypertension is a morbid condition that gained recognition in 1973 when the World Health Organization organized its first symposium. The hemodynamic definition was given as mean pulmonary artery pressure > 25 mmHg at rest measured by right heart catheterization [1]. The second symposium was marked by the classification of PH into five groups based on common pathophysiological findings. The association between pulmonary diseases and PH was thus formally established and assigned the third group in the classification. Dana Point Classification was given during the 4^th^ symposium and the treatment algorithm updated [2, 3]. In 2018, the 6^th^ World Symposium on Pulmonary Hypertension was a landmark as a proposal was made to revise hemodynamic definition and set mPAP threshold at a lower value to ≥20 mmHg [4, 5]. This proposal comes in light of the fact that normal mPAP is 14 mmHg ± 3.3 mmHg. Thus, the values from 20 to 25 mmHg remained a grey zone. Studies have proven that patients with mPAP 20–25 mmHg are likely to progress to ≥25 mmHg and are at higher risk for morbidity [4]. In our study, we aim to study the effect of the new definition on the prevalence of pulmonary among the patients of ILDs at our tertiary care centre.

## 2. Methods

This cross-sectional study was conducted in the Department of Respiratory Medicine, KGMU, UP, Lucknow, from 1^st^ September, 2018, to 31^st^ August, 2019. Consecutive patients diagnosed with interstitial lung diseases who provided written informed consent were included in the study. Approval was obtained from Institutional Ethics Committee. Patients who did not provide consent or had left-sided heart disease were excluded.

239 subjects were enrolled in the study. All were subjected to transthoracic echocardiography, and the following parameters were recorded: tricuspid regurgitation (TR) velocity, right ventricular systolic pressure (RVSP), pulmonary artery acceleration time, right atrial area, right atrial and right ventricular enlargement, tricuspid annular plane systolic excursion (TAPSE), fractional area change (FAC), interventricular septum (IVS) flattening, pericardial effusion, and left ventricular ejection fraction. Right atrial enlargement was said to occur when right atrial area > 18 cm^2^. TAPSE indicated the longitudinal function of right ventricle. Its normal value was >1.6 cm [6]. Mean pulmonary artery pressure was calculated using Mahan's equation: mPAP=79 − (0.45*∗*PAT) [7]. RVSP was calculated using TR velocity as RVSP = 4 V^2^ + right atrial pressure, where V is the TR velocity. The severity was defined as mild if mPAP was 20–40 mmHg in new definition or 25–40 mmHg in the previous one and moderate and severe when mPAP was 41–55 mmHg and >55 mmHg, respectively [8–10]. Diameter of right pulmonary artery > 16 mm on chest radiograph was used to define its enlargement [11]. Pro-B-type natriuretic peptide (pro-BNP) and troponin T levels were measured in all the subjects. The statistical analysis was done using SPSS (Statistical Package for Social Sciences) Version 21.0 statistical analysis software. The values were represented in number (%) and mean ± SD. The statistical tools employed were mean, standard deviation, chi-square test, Student' *t*-test, analysis of variance (ANOVA), and logistic regression.

## 3. Results

**Figure 1:**
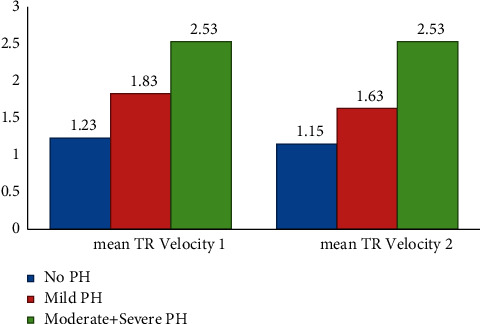
Mean TR velocity 1 (m/s) in subjects diagnosed with older definition and mean TR velocity 2 (m/s) in subjects diagnosed with newer definition.

**Figure 2:**
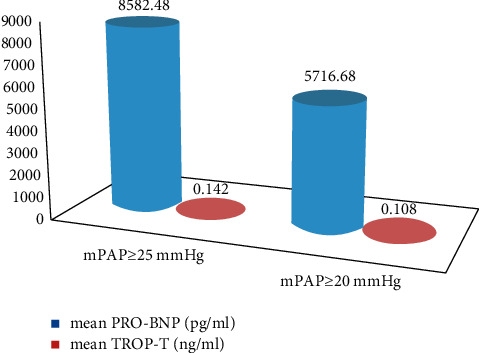
Mean pro-BNP and Trop-T values compared in the two groups.

**Figure 3:**
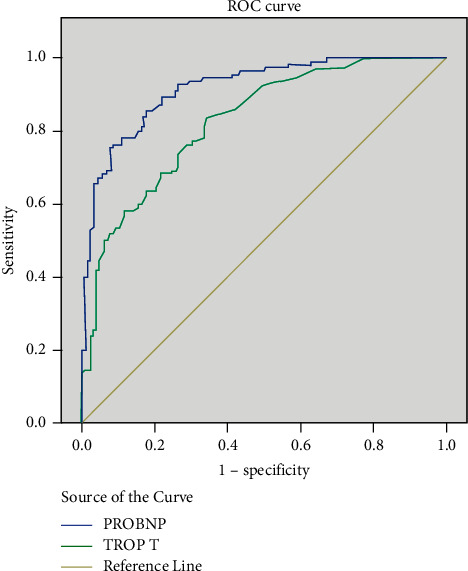
Prediction of pulmonary hypertension (≥20 mmHg) by serum pro-BNP and troponin T values (receiver-operating characteristic curve).

239 subjects were enrolled which comprised of females and had a mean age of years. Most were resident of rural region. Other baseline characteristics are given in Table 1. Majority of the patients fell under the diagnosis of hypersensitivity pneumonitis (HSP) and connective tissue disease-related interstitial lung disease (CTD-ILD). Rest were cases of idiopathic pulmonary fibrosis (IPF), non-IPF idiopathic interstitial pneumonias (IIPs), sarcoidosis, and others. The other ILDs included cases such as combined pulmonary fibrosis and emphysema (CPFE), Langerhans' cell histiocytosis (LCH), occupational lung disease, radiation-induced fibrosis, and unclassified. The distribution of population according to the ILD diagnosis is given in Table 2. When compared gender wise, IPF was more prevalent in males. All the subjects were subjected to transthoracic echocardiography, and statistics was applied using different cutoffs of mPAP. The prevalence of PH rose from 28.9% to 46.0% when mPAP > 20 mmHg was used to define PH (Tables 3 and 4). While the prevalence was higher in females, no statistical significance was seen using either definition. Among those with PH, nearly two-thirds were in mild category. The number of severe cases was only two; hence, it was combined with the moderate category while applying statistics. The largest number of cases was seen in HSP subjects, as it was the most prevalent group. Among the different types of ILDs, non-IPF IIPs had the maximum prevalence though the number of subjects was too small (Tables 5 and 6). Echocardiographic parameters were significantly deranged in the moderate-to-severe group only where mean tricuspid regurgitation velocity was 2.53 m/s^2^, mean RVSP was 54.7 mmHg, and mean diameter of IVC was 21.57 mm (Tables 7 and 8 and Figure 1). Chest radiograph findings were not so specific as reticulations are seen in ILDs as well. Pulmonary artery was enlarged only in moderate-to-severe cases. Right ventricular hypertrophy and p-pulmonale on ECG were also seen in moderate-severe category. In the lung function test, no significant correlation with was seen with forced vital capacity. Mean pro-BNP was 8582.48 pg/ml using the older definition group, while it decreased to 5716.68 pg/ml applying the new cutoff (Figure 2). We ascertained the level of pro-BNP and Trop-T above which all the subjects had mPAP ≥ 20 mmHg. Pro-BNP ≥ 535.5 pg/ml was 87.3% sensitive and 79.1% specific, while Trop-T ≥ 0.0295 ng/ml had sensitivity of 73.6% and specificity of 73.6% (Table 9 and Figure 3).

## 4. Discussion

The present study was carried out in the Department of Respiratory Medicine, King George's Medical University, Lucknow, for a duration of one year. The prevalence of PH was estimated among outdoor/indoor ILD patients according to both new and old definition. The lower cutoff of the new definition raised the prevalence by 17% among our patients.

Among the 239 ILD patients, hypersensitivity pneumonitis (*n* = 77) and CTD-ILD (*n* = 75) comprised most of the study group. Prevalence of other major groups is shown in Figure 1. The higher prevalence of these two subtypes is in accordance with ILD India registry that was the largest study on ILD in the country. The study population had an average age of 52.4 years with female predominance (Table 1). The prevalence of IPF was significantly higher in males (Table 2) as has been previously documented in different international as well as Indian studies [12–14]. While connective tissue disorders are more common in females, associated ILD has been reported more frequently in males [15, 16]. However, our study had a nearly equal prevalence that is probably due to an overall higher female proportion of the study population. When the older definition of PH was applied, 28.9% of the subjects surpassed the diagnostic cutoff (Table 3). Most fell in the range of mild PH, and only two qualified for the severe PH criteria. Applying the new criteria, the prevalence went up to comprise nearly half of the study population (Table 4). A higher prevalence in Indian scenario could possibly be attributed to a delay in diagnosis of ILD in the peripheral and rural areas that results in unchecked progression of the disease [17, 18]. Overall, the prevalence is higher in females, which is in accordance with the existing literature, and the values were not significant though [19, 20]. Female sex hormones have been implicated in the probable mechanism for a higher prevalence. Females have a higher number of estrogen receptors in all the tissues including blood vessels. In general, estrogen has the effect of boosting immunity and promotion of remodeling and fibrosis [21, 22]. The *β* type of estrogen receptors increases the arterial tone and raises the pressure, while the *α* type has a protective role and prevents fibrosis. The balance between these two types of receptors seems to be responsible for female preponderance [23, 24]. Both the subjects of severe PH are males; however, the number is too small to consider it relevant. The prevalence seems higher among IIPs other than IPF and other ILDs as the number of study subjects with these diagnosis was quite low. Nearly 18.2% of HSP patients had mild PH that rose to 37.7% with the revised definition (Tables 5 and 6) Although previous studies have utilized the old definition of PH to make the diagnosis, a high prevalence has been noted by many. Several studies have reported variable prevalence of pulmonary hypertension varying from 19% to 31% in cases of advanced IPF [25–27]. All have reported poorer outcome in patients with pulmonary hypertension. Gradually, the association of other ILDs with pulmonary hypertension was also recognized. A comparative study of pulmonary hypertension prevalence in IPF and NSIP was published in 2006 by Ahmad et al. reporting a poor outcome in the IPF [28]. Pulmonary hypertension is commonly associated with scleroderma and contributes to worse prognosis as has been reported in several studies by Mukerjee et al., Girgis et al., and Launay et al. [29–31]. In our study, out of 18 patients with CTD-ILD, 10 (55.55%) had systemic sclerosis.

A high prevalence has been reported previously in CPFE and other rare ILDs such as LCH and lymphangioleiomyomatosis in advanced cases [32–34]. Oliviera RK and Koschel DS have reported a fairly common prevalence of pulmonary hypertension in chronic hypersensitivity pneumonitis ranging from 19% to 50% [35, 36]. The current study has the highest number of HSP subjects which explains the higher number of PH cases in that group. Connective tissue diseases have a high prevalence of PH as has been demonstrated by several studies [37]. Shirai et al. found out that mixed CTD, systemic lupus erythematosus, and systemic sclerosis constituted 43%, 29%, and 19% of patients of CTD-ILD in their study, respectively [38]. Among Indian studies, Haroon et al. concluded that during early CTD, PH may not be present [39]. Our study shows a PH prevalence of 25.4% among CTD patients. Other studies have provided consolidating figures establishing the association of PH with CTD [40, 41].

In sarcoidosis, pulmonary hypertension has been reported in up to 20% of patients, and the mechanisms involved are different. Often, the ones with advanced disease are involved [42–44]. Concomitant vasculopathy and inflammatory milieu may also contribute to PH. In the present study, the prevalence of PH among sarcoidosis subjects varied from 33.4% (older definition) to 46.7% (new definition). Most patients report to our tertiary centre in fibrocystic stage in hypoxia, which can account for a higher prevalence of elevated pulmonary artery pressure.

The mean TR velocity in mild cases was 1.83 m/s, which decreased to 1.63 m/s on applying the new definition (Figure 2). Echocardiography and chest radiography showed significant aberration in moderate to severe PH patients (Tables 7 and 8). Previous studies vary on correlation of imaging findings with mean pulmonary artery pressure [45, 46]. Indeed, the diameter of the artery varies not just with the pressure but with blood flow as well. Mean pro-BNP and troponin T values were elevated in the subjects with PH (Table 9 and Figure 3). This is in accordance with American Heart Association guidelines [47]. We admit the drawback in our study that the gold standard investigation right heart catheterization was not done. There have been studies that have established the reliability of transthoracic echocardiography as a noninvasive diagnostic modality [48–50].

Thus, in our study, we could conclude that there were a considerable number of subjects with interstitial lung diseases who had their mPAP in the range of 20–25 mmHg, and with application of the new definition, the pulmonary hemodynamics got the attention earlier which is likely to impact the management and overall quality of life of such patients.

## 5. Conclusion

We have ascertained the prevalence of pulmonary hypertension among patients of interstitial lung diseases utilizing two different international definitions. The new definition certainly unmasks a large number of subjects who previously fell in the range of elevated pulmonary artery pressure but could not qualify the criteria for diagnosis.

## Figures and Tables

**Table 1:** Baseline characteristics of the study population (n = 239).

Characteristics	Statistics

Mean age ± SD (range) in years	52.4 ± 13.4 (15–88)
Gender	
Female	141 (59.0%)
Male	98 (41.0%)
Residence	
Rural	136 (56.9%)
Semiurban	2 (0.8%)
Urban	101 (42.3%)
Nonsmokers	188 (78.7%)
Smokers/exsmokers	51 (21.3%)
mPAP ± SD (range) in mmHg	22.3 ± 10.1 (8–61)
Mean pulmonary artery diameter ± SD (range) in mm	27.4 ± 3.4 (22–40)

SD, standard deviation; mPAP, mean pulmonary artery pressure.

**Table 2:** Distribution of subjects according to diagnosis.

S. no.	Final diagnosis	No. of patients	Females (*n* = 141)		Males (*n* = 98)	
			No.	%	No.	%

1	Hypersensitivity pneumonitis	77	45	58.4	32	41.6
2	CTD-ILDs	75	37	49.3	38	50.7
3	Sarcoidosis	30	14	46.7	16	53.3
4	IPF	38	11	28.9	27	71.1
5	Non-IPF IIPs	5	4	80.0	1	20.0
6	Others	14	7	50.0	7	50.0

%, row-wise; *χ*^2^ = 31.136 (df = 5); *p* < 0.001. CTD-ILDs, connective tissue disease related interstitial lung diseases; IPF, idiopathic pulmonary fibrosis; IIP, idiopathic interstitial pneumonia; df, degrees of freedom.

**Table 3:** Prevalence and severity of pulmonary hypertension (mPAP ≥ 25 mmHg).

S. no.	Severity of pulmonary hypertension	Number of patients	Percentage	Female (*n* = 141)		Male (*n* = 98)	
				No.	%	No.	%

1	No pulmonary hypertension	170	71.1	98	57.6	72	42.4
2	Mild pulmonary hypertension	39	16.3	26	66.7	13	33.3
3	Moderate pulmonary hypertension	28	11.7	17	60.7	11	39.3
4	Severe pulmonary hypertension	2	0.8	0	0.0	2	100.0

*χ* ^2^ = 3.988 (df = 3); *p*=0.263; *χ*^2^ = 0.443 (df = 1); *p*=0.506 (for no pulmonary hypertension (*n* = 170) vs. pulmonary hypertension (*n* = 69)). mPAP, mean pulmonary artery pressure; df, degrees of freedom.

**Table 4:** Prevalence and severity of pulmonary hypertension (mPAP ≥ 20 mmHg).

S. no.	Severity of pulmonary hypertension	Number of patients	Percentage	Female (*n* = 141)		Male (*n* = 98)	
				No.	%	No.	%

1	No pulmonary hypertension	129	54.0	72	55.8	57	44.2
2	Mild pulmonary hypertension	80	33.5	52	65.0	28	35.0
3	Moderate pulmonary hypertension	28	11.7	17	60.7	11	39.3
4	Severe pulmonary hypertension	2	0.8	0	0.0	2	100.0

*χ* ^2^ = 4.644 (df = 3); *p*=0.200; *χ*^2^ = 1.173 (df = 1); *p*=0.279 (for no pulmonary hypertension (*n* = 129) vs. pulmonary hypertension (*n* = 110)). mPAP, mean pulmonary artery pressure; df, degrees of freedom.

**Table 5:** Diagnosis-wise prevalence and severity of pulmonary hypertension (mPAP ≥ 25 mmHg).

Diagnosis	Total	No pulmonary hypertension (*n* = 170)		Mild pulmonary hypertension (*n* = 39)		Moderate + severe pulmonary hypertension (*n* = 30)	
		No.	%	No.	%	No.	%

HP	77	52	67.5	14	18.2	11	14.3
CTD-ILD	75	56	74.7	11	14.7	8	10.7
Sarcoidosis	30	20	66.7	5	16.7	5	16.7
IPF	38	31	81.6	2	5.3	5	13.2
Non-IPF IIP	5	4	80.01	1	20.0	0	0.0
Others	14	7	50.0	6	42.9	1	7.1

*χ* ^2^ = 12.862; *p*=0.232. CTD-ILD, connective tissue disease-related interstitial lung disease. IPF, idiopathic pulmonary fibrosis; IIP, idiopathic interstitial pneumonia.

**Table 6:** Diagnosis-wise prevalence and severity of pulmonary hypertension (mPAP ≥ 20 mmHg).

Diagnosis	Total, *n* = 239	No pulmonary hypertension (*n* = 110)		Mild pulmonary hypertension (*n* = 75)		Moderate + severe pulmonary hypertension (*n* = 30)	
		No.	%	No.	%	No.	%

HP	77	37	48.1	29	37.7	11	14.3
CTD-ILD	75	42	56.0	25	33.3	8	10.7
Sarcoidosis	30	19	63.3	6	20.0	5	16.7
IPF	38	20	52.6	13	34.2	5	13.2
Non-IPF IIP	5	4	80.0	1	20.0	0	0.0
Others	14	7	50.0	6	42.9	1	7.1

*χ* ^2^ = 6.168; *p*=0.801. CTD-ILD, connective tissue disease-related interstitial lung disease; IPF, idiopathic pulmonary fibrosis; IIP, idiopathic interstitial pneumonia.

**Table 7:** Association of pulmonary hypertension (mPAP ≥ 25 mmHg) and transthoracic echocardiography, ECG, and chest radiograph findings.

Variable	No pulmonary hypertension (*n* = 170)		Mild pulmonary hypertension (*n* = 39)		Moderate + severe pulmonary hypertension (*n* = 30)		ANOVA	
	Mean	SD	Mean	SD	Mean	SD	F	*P*

TR velocity (m/s)	1.23	0.38	1.83	0.80	2.53	1.06	63.642	<0.001
RSVP (mmHg)	26.66	5.50	42.90	5.76	54.70	13.55	249.880	<0.001
Pulmonary AT (ms)	67.68	12.41	49.24	3.35	35.85	7.42	134.256	<0.001
IVC diameter (mm)	14.09	1.18	17.90	2.14	21.57	1.98	375.356	<0.001
RA area (cm^2^)	9.52	2.10	14.86	3.79	17.40	3.63	151.420	<0.001
TAPSE (cm)	2.14	0.15	1.88	0.24	1.89	0.39	37.242	<0.001
FAC (%)	38.71	5.15	41.59	7.13	39.10	6.71	4.024	0.019
LVEF (%)	61.26	2.86	60.92	3.65	59.43	4.84	3.932	0.021
	No.	%	No.	%	No.	%	*χ* ^2^	*P*
Right atrial enlargement	0	0.0	11	28.2	20	66.7	109.981	<0.001
Right ventricular enlargement	0	0.0	7	17.9	19	63.3	107.902	<0.001
Small left chamber	0	0.0	0	0.0	0	0.0	—	—
IV septum flattening	1	0.6	1	2.6	2	6.7	5.950	0.051
Pericardial effusion	0	0.0	0	0.0	1	3.3	6.996	0.030
Chest X-ray								
Reticulations	24	14.1	3	7.7	7	23.3	3.405	0.182
Cardiomegaly	0	0.0	0	0.0	9	30.0	65.153	<0.001
Enlarged PA	0	0.0	1	2.6	5	16.7	28.942	<0.001
Pruning	0	0.0	0	0.0	1	3.3	6.996	0.030
RVH	0	0.0	1	2.6	17	56.7	119.229	<0.001
RAD	0	0.0	1	2.6	21	70.0	151.962	<0.001
P-pulmonale	0	0.0	1	2.6	13	43.3	87.747	<0.001

TR, tricuspid regurgitation; RVSP, right ventricular systolic pressure; AT, acceleration time; IVC, inferior vena cava; RA, right atrium; TAPSE, tricuspid annular plane systolic excursion; FAC, fractional area change; LVEF, left ventricular ejection fraction; RV, right ventricle; IVS, interventricle septum; PA, pulmonary artery; ECG, electrocardiogram; RVH, right ventricular hypertrophy; RAD, right axis deviation.

**Table 8:** Association of pulmonary hypertension (≥20 mmHg) and 2D echo findings, ECG, and X-ray findings.

Findings	No pulmonary hypertension (*n* = 129)		Mild pulmonary hypertension (*n* = 80)		Moderate + severe pulmonary hypertension (*n* = 30)		ANOVA	
	Mean	SD	Mean	SD	Mean	SD	F	*P*

TR velocity	1.15	0.28	1.63	0.69	2.53	1.06	64.863	<0.001
RSVP	24.84	4.06	37.50	7.73	54.70	13.55	236.263	<0.001
Pulmonary AT	70.00	13.39	54.95	6.30	35.85	7.42	137.854	<0.001
IVC diameter	13.91	1.04	16.25	2.41	21.57	1.98	244.722	<0.001
RA area (cm^2)^	9.05	1.74	12.88	3.71	17.40	3.63	124.899	<0.001
TAPSE	2.14	0.12	2.01	0.26	1.89	0.39	19.514	<0.001
FAC (%)	38.76	5.34	40.04	6.11	39.10	6.71	1.212	0.299
LVEF (%)	61.26	2.42	61.11	3.81	59.43	4.84	3.804	0.024
		%	No.	%	No.	%	*χ* ^2^	*P*
RA enlargement	0	0.0	11	13.8	20	66.7	95.895	<0.001
RV enlargement	0	0.0	7	8.8	19	63.3	101.260	<0.001
Small left chamber	0	0.0	0	0.0	0	0.0	—	—
IV septum flattening	0	0.0	2	2.5	2	6.7	7.073	0.029
Pericardial effusion	0	0.0	0	0.0	1	3.3	6.996	0.030
Chest X-ray								
Reticulations	17	13.2	10	12.5	7	23.3	2.351	0.309
Cardiomegaly	0	0.0	0	0.0	9	30.0	65.153	<0.001
Enlarged PA	0	0.0	1	1.3	5	16.7	28.405	<0.001
Pruning	0	0.0	0	0.0	1	3.3	6.996	0.030
RVH	0	0.0	1	1.3	17	56.7	119.041	<0.001
RAD	0	0.0	1	1.3	21	70.0	151.805	<0.001
P-pulmonale	0	0.0	1	1.3	13	43.3	87.508	<0.001

TR, tricuspid regurgitation; RVSP, right ventricular systolic pressure; AT, acceleration time; IVC, inferior vena cava; RA, right atrium; TAPSE, tricuspid annular plane systolic excursion; FAC, fractional area change; LVEF, left ventricular ejection fraction; RV, right ventricle; IVS, interventricle septum; PA, pulmonary artery; ECG, electrocardiogram; RVH, right ventricular hypertrophy; RAD, right axis deviation.

**Table 9:** Prediction of pulmonary hypertension (≥20 mmHg) by serum pro-BNP and troponin T values (Figure 3: ROC curve).

Test result variables	Area	Standard error (a)	Asymptotic sig.(b)	Asymptotic 95% confidence interval	
				Lower bound	Upper bound

Pro-BNP	0.918	0.017	<0.001	0.884	0.952
Trop-T	0.828	0.026	<0.001	0.777	0.878

## Data Availability

The data used to support the findings of this study are available from the corresponding author upon request.
